# The curse of the teenage learner

**DOI:** 10.1111/medu.12405

**Published:** 2014-02-16

**Authors:** Liz Mossop

**Affiliations:** NottinghamUK

The Hollywood film *Field of Dreams* depicts a farmer who dreams of hosting professional baseball games on his land. As he walks through his fields of corn, a ghostly voice, referring to the farmer's imagined baseball field, the star player and the spectators who will consequently flock to the site, calls: ‘If you build it, he will come.’

As clinical educators, we make similar assumptions all the time. We want to do what is best for our learners. We assume that if we invest time, energy and money into developing an interactive and engaging curriculum, students will attend. Not only will they attend, but they will also have completed the pre-sessional tasks we have set them, and will focus on their learning during the session, abandoning the distractions of social media and their peers. Why wouldn't they do this? They want to be clinicians, so surely they recognise the need to come to teaching sessions and engage with faculty staff?

We assume that if we invest time, energy and money into developing an interactive and engaging curriculum, students will attend.

Unfortunately, the frank and honest paper by White *et al*.^1^ published in this issue of the journal, shows that our best efforts to produce a student-centred curriculum to encourage active learning and engagement can sometimes fail to deliver the experience we predicted. In this example of an obviously genuine attempt to deliver a modern new curriculum with a flipped classroom approach, student attendance dropped as low as 25%.^1^ It is easy to picture a faculty meeting at this institution, at which frustrated voices cite the wasting of resources and the pointless implementation of new methods of delivery. Why should we bother taking new approaches when students fail to fulfil their obligations as learners? Surely it would be sensible to retain the ‘safe option’ of didactic delivery?

Why should we bother taking new approaches when students fail to fulfil their obligations as learners?

Rather than allowing the effort put into making these changes to go to waste, White *et al*.^1^ have taken time to consider the reasons for this disengagement. Their conclusions are very useful: they suggest that students need better training to learn effectively this way, and that faculty development is also required. However, it is also essential to consider the culture in which this change was attempted.

The learning culture has much to answer for when curricular changes are made. Although it is true that students entering undergraduate training may not be ready for a more ‘grown-up’ form of learning, and may not yet possess the self-direction and self-motivation they require for success, there may also be unseen influences that prevent successful delivery. Impressionable teenage learners, who have not yet learned the skills of adult learning, are not only exposed to the formal curriculum we deliver, but also to the unseen influences of peer role models and rituals within the hidden curriculum.^2^ These latter influences may pull them away from their core activities of study and learning, distracting them from their central focus of learning to be a clinician. A change in learning culture is therefore necessary in order to implement new teaching strategies such as those involved in the flipped classroom^3^ and, furthermore, both students and faculty members need help to engage effectively in this different culture.

Impressionable teenage learners are not only exposed to the formal curriculum, but also to the unseen influences of peer role models and rituals within the hidden curriculum.

Interestingly, the paper by White *et al*.^1^ notes the supportive nature of students' interactions with one another, which emerged strongly in student focus groups engaged in discussing the curriculum. Although this is an attribute we should encourage in students as a positive behaviour during the stage of proto-professionalism prior to their emerging as professionals,^4^ it does not in this context help their learning. As well as training students in active learning techniques, we should facilitate explicit discussion of the influence of the hidden curriculum as this will help students to understand how elements such as negative role models can influence their learning behaviours.^5^ Students should also be encouraged to reflect on their learning styles and abilities in order to develop as learners who continue to help each other whilst maintaining their focus on the information they are considering. They should be explicitly taught the process of reflection because this is a required attribute for success as a student in a curriculum that embraces active learning approaches.^6^ Reflective skills will also be necessary as an aspect of future professionalism and thus their development at a very early stage is extremely useful.^7^,^8^

Students should be encouraged to reflect on their learning styles and abilities in order to develop as learners.

It is somewhat disappointing, perhaps, that the faculty staff referred to in this study^1^ did not consider the learning culture before investing time and money in a new curriculum. Whenever curricula are planned, faculty staff must perform a thorough needs assessment,^9^ which should include an analysis of teaching delivery aligned to learners' ability and faculty staff engagement. It is easy to read about initiatives such as that of flipping the classroom and start to embed them in a redesigned curriculum without considering issues such as learner readiness and institutional culture. New delivery methods such as this may have limited contextual evidence to show their effectiveness,^3^ but lessons learned from similar strategies involving active learning can certainly be drawn from. Problem-based learning curricula have certainly been well researched and there is plenty of evidence to show that their failure often derives from a lack of faculty readiness, student disengagement and the powerful influence of the hidden curriculum.^10^,^11^

It is easy to read about initiatives such as that of flipping the classroom and start to embed them in a redesigned curriculum without considering learner readiness and institutional culture.

It is therefore important that curriculum designers reflect thoroughly on all aspects of institutional culture before implementing new and innovative teaching strategies. What works in one environment may be entirely ineffective in another context. Faculty staff must be mindful of the ‘curse of the teenage learner’ and must be ready to teach students to learn, as well as to recognise the influence of their peers as role models. Only then will active learning become embedded in the culture and practices of learning in an institution, and the hidden curriculum will become an ally rather than an enemy. Only then may the field of dreams become a reality for clinical educators.
